# Metformin Overcomes Acquired Resistance to EGFR TKIs in EGFR-Mutant Lung Cancer via AMPK/ERK/NF-κB Signaling Pathway

**DOI:** 10.3389/fonc.2020.01605

**Published:** 2020-09-10

**Authors:** Ling Li, Tao Wang, Mengdi Hu, Yali Zhang, Hongzhuan Chen, Lu Xu

**Affiliations:** ^1^Department of Pharmacology and Chemical Biology, Shanghai Jiao Tong University School of Medicine, Shanghai, China; ^2^Institute of Interdisciplinary Integrative Biomedical Research, Shanghai University of Traditional Chinese Medicine, Shanghai, China

**Keywords:** metformin, lung cancer, EGFR TKIs, acquired resistance, NF-κB, AMPK

## Abstract

**Background:** The major limitation of EGFR TKIs in EGFR-mutant lung cancer therapy is the development of acquired resistance. The underlying mechanisms remain unknown in about 30% of cases. NF-κB activation was encountered in the acquired resistance to EGFR TKIs. Unfortunately, none of NF-κB inhibitors has been clinically approved. The most commonly used antidiabetic drug metformin has demonstrated antitumor effects associated with NF-κB inhibition. Therefore, in this study, metformin was examined for its antitumor and antiresistance effects and underlying mechanisms.

**Methods:** *In vitro* and *in vivo* EGFR-mutant lung cancer models with acquired resistance to EGFR TKIs were used.

**Results:** We found that NF-κB was activated in EGFR-mutant lung cancer cells with acquired resistance to EGFR TKIs. Metformin inhibited proliferation and promoted apoptosis of lung cancer cells, especially those with acquired EGFR TKI resistance. Moreover, metformin reversed and delayed acquired resistance to EGFR TKIs as well as suppressed cancer stemness in EGFR-mutant lung cancer. Mechanistically, those effects of metformin were associated with activation of AMPK, resulting in the inhibition of downstream ERK/NF-κB signaling.

**Conclusions:** Our data provided novel and further molecular rationale and preclinical data to support combination of metformin with EGFR TKIs to treat EGFR-mutant lung cancer patients, especially those with acquired resistance.

## Background

EGFR tyrosine kinase inhibitors (TKIs) have demonstrated dramatic efficacy in non-small cell lung cancer (NSCLC) patients with EGFR-activating mutations (1). Despite impressive initial responses, almost all patients eventually have relapses due to the occurrence of acquired resistance. The secondary EGFR mutation has been reported to be the most common mechanism of acquired resistance to TKIs, such as T790M after first-generation TKIs and T797S after third-generation TKI osimertinib (2, 3). The other mechanisms of acquired resistance including bypass or downstream activation (MET amplification, PIK3CA mutation, and AXL activation) and histological transformation have been reported (4, 5). However, the mechanisms remain unknown in about 30% of cases. Since the clinical efficacy of TKIs is ultimately limited by the development of acquired resistance, further investigation of novel molecular mechanisms is essential to develop strategies to overcome or delay the acquired resistance to TKIs in EGFR-mutant lung cancer.

The nuclear factor-κB (NF-κB), a transcription factor that is essential in the regulation of immune responses and inflammation, has been recognized as having important roles in cancer development and progression in the past few decades. Aberrant or constitutive NF-κB activation has been encountered in many cancers including both solid and hematopoietic malignancies (6–8). In addition to promoting cancer cell proliferation and survival, NF-κB activation is also involved in epithelial-to-mesenchymal transition (EMT), invasion, angiogenesis, metastasis, stemness acquisition, and therapeutic resistance (9–11). We and others have previously reported that NF-κB was activated in acquired resistance to EGFR TKIs in *in vitro* and *in vivo* models and patient samples of EGFR-mutant lung cancer (12–14). Therefore, NF-κB is widely considered a potential therapeutic target (15). However, the complex interaction of the NF-κB signaling network with other oncogenic pathway presents challenges for specifically targeting NF-κB (16). Despite the aggressive efforts to develop NF-κB inhibitors, none has been clinically approved, mostly due to immune-related toxicities associated with global NF-κB suppression (17).

Since none of newly developed NF-κB inhibitors have been successfully translated into clinic, we have looked at old drugs to find new indications. Metformin is an oral antidiabetic drug that has been safely used worldwide for decades. Daily intake of 250–2,000 mg metformin has been linked to a decreased risk and mortality of several cancers (18). However, the anticancer mechanisms of metformin, the most commonly used drug and promising candidate of drug repositioning, have not been clear yet. Several studies have demonstrated that anticancer effects of metformin have been associated with NF-κB signaling inhibition (19, 20). The aim of our study was to investigate whether metformin could inhibit NF-κB signaling in EGFR-mutant lung cancer, and if so, whether it could have anticancer effects on lung cancer and anti-resistance efficacy against acquired EGFR TKIs resistance and underlying mechanisms. In this study, we have first established *in vitro* and *in vivo* EGFR-mutant lung cancer models with acquired resistance to EGFR TKIs, including the third generation TKI osimertinib. Then, we reported that metformin selectively inhibited proliferation and promoted apoptosis of lung cancer cells especially resistant cells due to their constitutive and increased NF-κB activation. Moreover, metformin significantly delayed and reversed acquired resistance to EGFR TKIs as well as suppressed lung cancer stemness. Mechanistically, those effects of metformin were associated with activation of AMPK, resulting in the inhibition of downstream ERK/NF-κB signaling. Taken together, our data demonstrate metformin as a potential candidate for combination therapy for EGFR-mutant lung cancer.

## Materials and Methods

### Chemicals

Metformin and TNFα were purchased from Sangon Biotech (Shanghai, China) and dissolved in sterile distilled water. Gefitinib and osimertinib were purchased from MedChemExpress (Shanghai, China) and dissolved in DMSO. AICAR (5-aminoimidazole-4-carboxamide-1-β-D-ribofuranoside) was purchased from MedChemExpress and dissolved in sterile distilled water.

### Cell Culture and Establishment of Resistant Cancer Cell Lines *in vitro*

HCC827 and 16HBE were purchased from Cell Bank of Type Culture Collection of the Chinese Academy of Sciences (Shanghai, China) and PC9 cells were gifts from Dr. Qianggang Dong in Shanghai Cancer Institute, Shanghai Jiao Tong University School of Medicine. The cell lines were cultured under standard condition and tested by certified third-party laboratories for authenticity using short tandem repeat analysis. Gefitinib- and osimertinib-resistant HCC827 (HCC827GR and HCC827OR) and PC9 (PC9GR and PC9OR) cells were established by the stepwise escalation method and maintained as previously described (12).

### Cell Proliferation Assay

Cell proliferation was determined by cell viability using the Cell Counting Kit-8 (CCK-8) colorimetric assay (Dojindo, Shanghai, China) and the IncuCyte ZOOM® system (Essen BioScience) as previously described (12).

### Western Blot and Immunofluorescence Analyses

Western blot and immunofluorescence staining analyses were used to examine the expression levels of proteins as previously described (12). The list of used antibodies is in the Table S1. A Leica SP8 confocal microscope was used to acquire images using a 40× or 63× objective. Nuclear and cytoplasmic staining intensity were compared to give the nuclear/cytoplasmic ratio as a relative measure of p65 nuclear localization as previously described (21).

### Reporter Constructs and Dual-Luciferase Assay

The NF-κB response element (GGGAATTTCCGGGAATTTCCGGGAATTTCCGGGAATTTCC) was cloned into NheI site of psiCHECK-2 (Promega) vector to construct psiCHECK-NF-κB and verified by sequencing. Dual-Luciferase Reporter Assay System (Promega) was used to measure luciferase activity according to the manufacturer's manual.

### Colony Formation Assay

A total of 800–1,000 viable cells were seeded in six-well plates and cultured in complete medium for about 2–3 weeks. Colonies were fixed and stained with crystal violet.

### Mouse Xenograft Models, Combination Treatment, and Tumorigenic Assay

Athymic BALB/c nude mice were purchased from Shanghai Laboratory Animal Center (Chinese Academy of Sciences, Shanghai, China) and housed in environmentally controlled, specific pathogen–free conditions for 10 days before the study. All experimental procedures were reviewed and approved in accordance with the guidelines for the care and use of laboratory animals at Shanghai Jiao Tong University.

To establish xenograft models, the same amount of tumor cells was injected subcutaneously into both flanks of each mouse. The tumor volume was measured after 10 days from injection and then twice a week. Tumor volumes (mm^3^) were calculated as length × width^2^/2.

For treatment experiments, when xenograft tumors reached ~200 mm^3^, mice were given PBS, gefitinib (12.5 mg/kg), metformin (200 mg/kg), or a combination of gefitinib (12.5 mg/kg) and metformin (200 mg/kg) by oral gavage daily.

For tumorigenic assay, cells were treated with metformin (2 mM) for 12 h and then viable cells (1 × 10^6^, 5 × 10^5^, 2 × 10^5^, and 1 × 10^5^) in 50 μl of PBS were injected subcutaneously into each mouse. Xenograft tumor initiation and growth were examined twice a week.

### Statistical Analysis

All data are presented as the mean ± SEM. Statistical analysis was conducted using GraphPad Prism 7.0 software (La Jolla, CA, USA). Differences between groups were examined using Student's *t*-test or one-way ANOVA. Differences were considered significant if *P* < 0.05.

## Results

### Metformin Inhibited NF-κB Activity in Lung Cancer Cells

**Figure 1:**
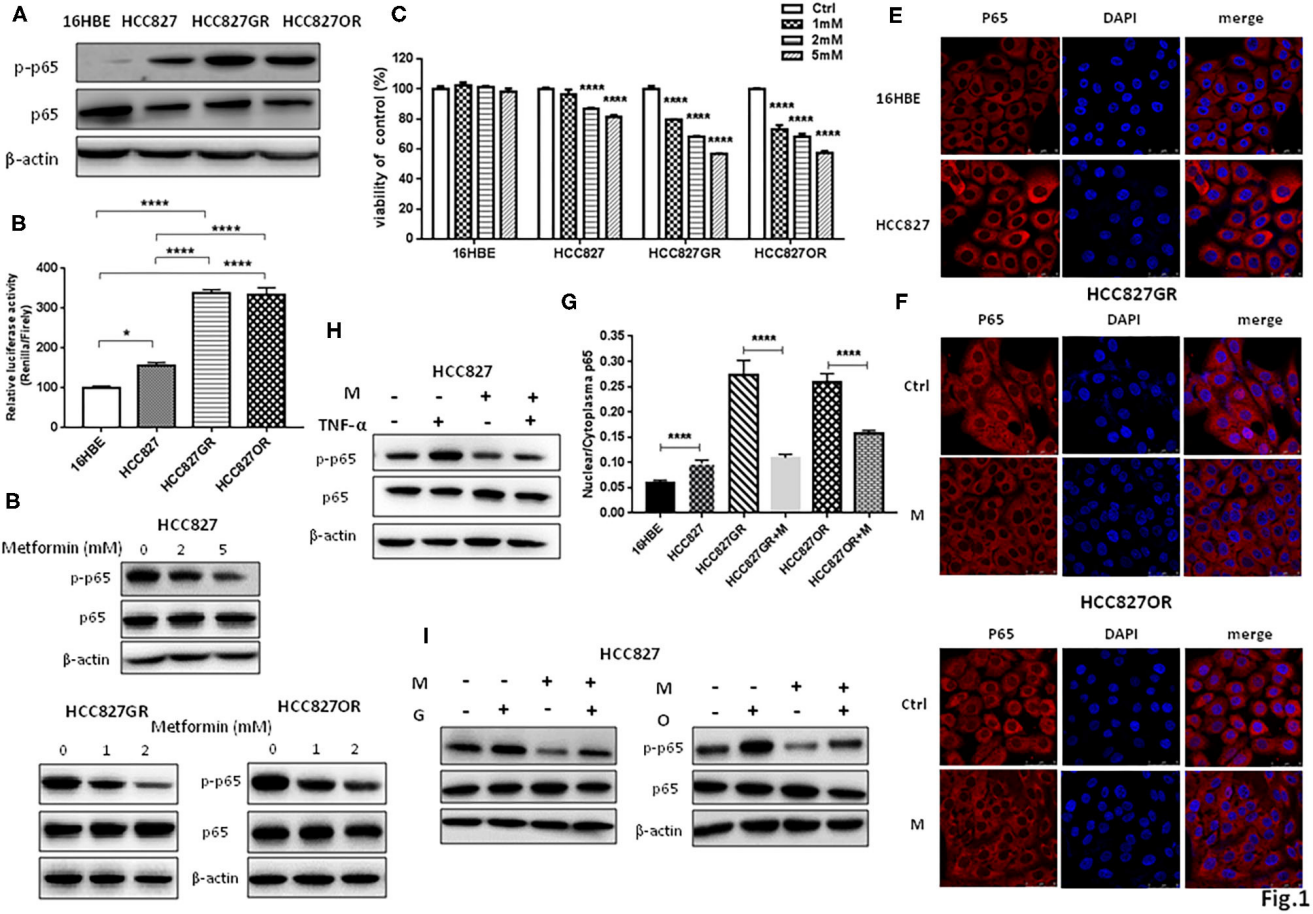
Metformin inhibited NF-κB activity in lung cancer. **(A)** Phosphorylated NF-κB p65 (p-p65) and NF-κB p65 (p65) in 16HBE, HCC827, HCC827GR, and HCC827OR cells were detected by Western blot. **(B)** Relative luciferase activity of indicated cell lines transiently transfected with psiCHECK-NF-κB. Results show the means ± SEM (*n* = 6, one-way ANOVA, *P < 0.05; ****P < 0.0001). **(C)** Cell viability by CCK8 assay of indicated cell lines treated with indicated concentrations of metformin for 72 h. Results show the means ± SEM (*n* = 6, one-way ANOVA, ****P < 0.0001). **(D)** Western blot of p-p65 and p65 in HCC827, HCC827GR, and HCC827OR cells treated with indicated concentrations of metformin (mM) for 72 h. **(E)** Immunofluorescence staining of p65 in 16HBE and HCC827 cells. Nuclei were stained with DAPI. Scale bar, 50 μm. **(F)** Immunofluorescence staining of p65 in HCC827GR and HCC827OR cells treated with 1 mM metformin (M) for 72 h. Nuclei were stained with DAPI. Scale bar, 50 μm. **(G)** Quantitative analysis of **(E,F)**. Results show the means ± SEM (*n* = 3, Student's *t*-test, ****P < 0.0001). **(H)** Western blot of p-p65 and p65 in HCC827 cells pretreated with 2 mM metformin (M) for 70 h and then subjected to treatment of TNF-α (10 ng/ml) for 2 h. **(I)** Western blot of p-p65 and p65 in HCC827 cells pre-treated with 2 mM metformin (M) for 2 h and then subjected to treatment of 10 nM osimertinib (O) or 10 nM gefitinib (G) for 70 h.

We and others have previously reported that NF-κB was activated in EGFR-mutant lung cancer with acquired resistance to EGFR TKIs (12–14). *In vitro* cell model of acquired resistance to EGFR TKIs was established by culturing sensitive EGFR-mutant lung cancer HCC827 or PC9 cells in gefitinib or osimertinib with elevating concentration as we previously reported (12). Consistently, Western blot in Figure 1A and Figure S1A showed that acquired resistant HCC827GR and HCC827OR cells had increased levels of phosphorylated NF-κB p65 (p-p65) than parental HCC827 cells. Moreover, normal lung epithelial cells 16HBE had the lowest levels of p-p65 among HCC827, HCC827GR, and HCC827OR cells. NF-κB activity was also measured by luciferase activity by transiently transfecting cells with a dual-luciferase vector containing a Renilla luciferase gene under the control of NF-κB responsive element and a minimal promoter (psiCHECK-NF-κB). As expected, relative luciferase activity of 16HBE cells transiently transfected with psiCHECK-NF-κB was the lowest, while that of HCC827GR or HCC827OR was the highest (Figure 1B). The levels of phosphorylated NF-κB p65 (p-p65) were also dramatically increased in another EGFR-mutant lung cancer cell line with acquired resistance to EGFR TKIs (PC9GR and PC9OR) compared to parental/sensitive cell line PC9, as shown in Figure S2, demonstrating that NF-κB was activated in EGFR-mutant lung cancer with acquired resistance to EGFR TKIs.

Next, we treated those cell lines with increasing concentrations of metformin for 72 h and then examined cell viability by CCK8 assay to determine the effective concentration of metformin. Metformin had no inhibitory effects on 16HBE cell viability at 1, 2, and 5 mM. On the contrary, metformin had significant inhibitory effects on parental HCC827 cells at 2 and 5 mM and on resistant cells HCC827GR and HCC827OR in a concentration-dependent manner at 1, 2, and 5 mM (Figure 1C). Therefore, metformin at 1 and 2 mM concentrations was chosen to treat resistant cells and metformin at 2 and 5 mM was chosen to treat sensitive cells in the following experiments. As expected, metformin suppressed NF-κB activity by decreasing p-p65 levels (Figure 1D and Figure S1B) and p65 nuclear translocation (Figures 1E–G). Moreover, HCC827 cells were treated with NF-κB activator TNF-α with or without metformin. As shown in Figure 1H and Figure S1C, TNF-α increased phosphorylated NF-κB p65 levels while metformin abrogated TNF-α-induced p65 phosphorylation. Metformin also abolished gefitinib- and osimertinib-induced p65 phosphorylation (Figure 1I and Figure S1D), further demonstrating metformin suppressed NF-κB activity in EGFR-mutant lung cancer.

### Metformin Inhibited Lung Cancer Cell Proliferation and Promoted Apoptosis

**Figure 2:**
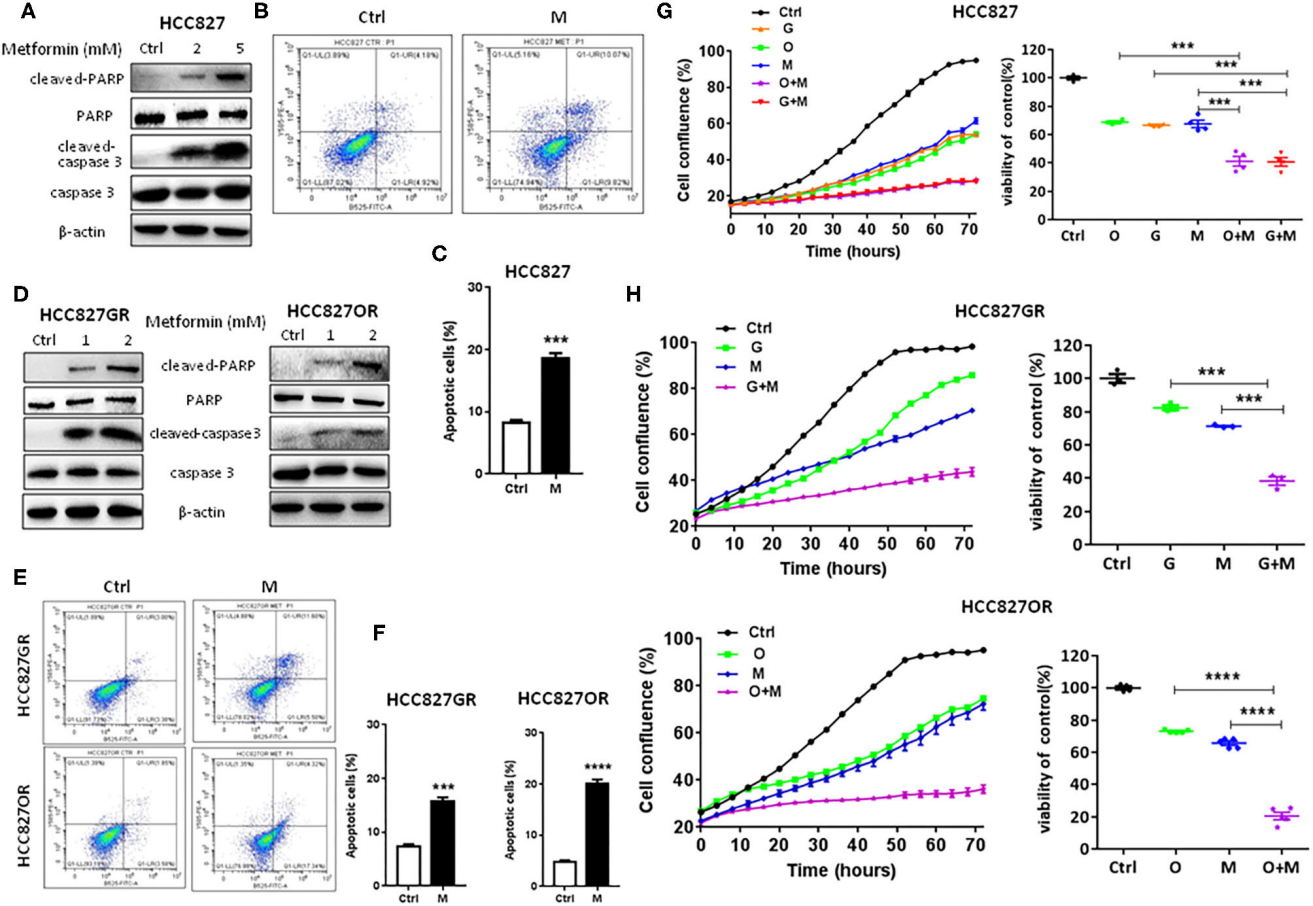
Metformin promoted apoptosis and inhibited proliferation of lung cancer cells. **(A)** Western blot of cleaved-caspase 3, caspase 3, cleaved-PARP, and PARP in HCC827 cells treated with indicated concentrations (mM) of metformin for 72 h. **(B)** Flow cytometry analysis of apoptosis of HCC827 cells treated with 2 mM metformin (M) for 72 h. **(C)** Quantitative analysis of apoptotic cells in **(B)**. Results show the means ± SEM (*n* = 3, Student's *t*-test, ***P < 0.001). **(D)** Western blot of cleaved-caspase 3, caspase 3, cleaved-PARP, and PARP in HCC827GR and HCC827OR cells treated with indicated concentrations (mM) of metformin for 72 h. **(E)** Flow cytometry analysis of apoptosis of HCC827GR and HCC827OR cells treated with 1 mM metformin (M) for 72 h. **(F)** Quantitative analysis of apoptotic cells in **(E)**. Results show the means ± SEM (*n* = 3, Student's *t*-test, ***P < 0.001, ****P < 0.0001). **(G)** HCC827 cells were treated with 10 nM osimertinib (O), 10 nM gefitinib (G), 2 mM metformin (M), O+M or G+M. IncuCyte growth curve (left panel) and cell viability by CCK8 assay (right panel, 72 h). Results show the means ± SEM (*n* = 6 for IncuCyte; *n* = 4 for CCK8, one-way ANOVA, ***P < 0.001). **(H)** HCC827GR and HCC827OR cells were treated with 10 μM gefitinib (G), 10 μM osimertinib (O), 1 mM metformin (M), O+M or G+M. IncuCyte growth curves (left panels) and cell viabilities by CCK8 assay (right panels, 72 h). Results show the means ± SEM (*n* = 6 for IncuCyte; *n* = 3–5 for CCK8, one-way ANOVA, ***P < 0.001, ****P < 0.0001).

Since metformin suppressed NF-κB activity, next we tested its effects on lung cancer cell proliferation and apoptosis. As shown in Figures 2A–F and Figures S3A,B, in a concentration-dependent manner, metformin (M) promoted apoptosis of parental/sensitive HCC827 and EGFR TKI-acquired resistant HCC827GR and HCC827OR cells by upregulating cleaved-PARP and cleaved-caspase-3 expression (Figures 2A,D and Figures S3A,B) and increasing percentage of apoptotic cells by flow cytometry analysis (Figures 2B–F). Moreover, IncuCyte growth curves (left panels of Figures 2G,H) and cell viability assays (right panels of Figures 2G,H) both demonstrated that metformin (M) significantly inhibited proliferation of both parental/sensitive HCC827 and EGFR TKI-acquired resistant HCC827GR and HCC827OR. Similar results were observed using parental/sensitive PC9 and PC9OR cells (Figures S4A,B).

### Metformin Overcame and Delayed Acquired EGFR TKIs Resistance in Lung Cancer

**Figure 3:**
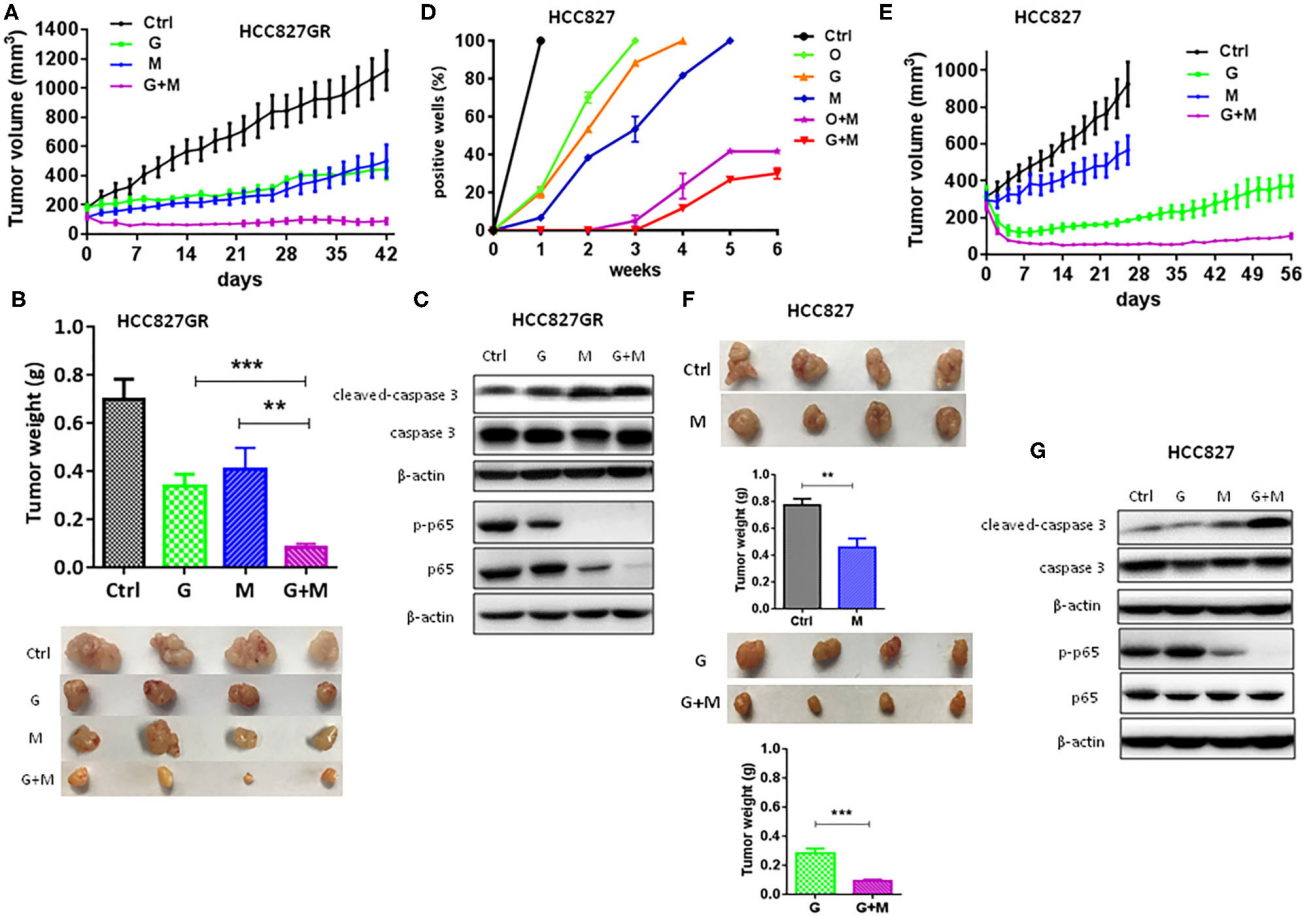
Metformin overcame and delayed acquired resistance to EGFR TKIs in lung cancer. **(A–C)** HCC827GR xenograft tumors were treated with PBS, gefitinib (G, 12.5 mg/kg), metformin (M, 200 mg/kg), or G+M daily by oral gavage. The growth curve **(A)**, and the weight and photographs of tumors **(B)**. Results show the means ± SEM (*n* = 4, one-way ANOVA, **P < 0.01, ***P < 0.001). **(C)** Western blot of cleaved-caspase 3, caspase 3, p-p65, and p65. **(D)** A total of 500 viable HCC827 cells were seeded per well in a 96-well plate and treated with O (10 nM), G (10 nM), M (2 mM), G+M, or O+M, and wells with >50% confluence were scored as positive weekly. Results show the means ± SEM (*n* = 10). **(E–G)** HCC827 xenograft tumors were treated with PBS, gefitinib (G, 12.5 mg/kg), metformin (M, 200 mg/kg), or G+M daily by oral gavage. The growth curve **(E)**, and the weight and the photographs of tumors **(F)**. Results show the means ± SEM (*n* = 4, Student's *t*-test, **P < 0.01, ***P < 0.001). **(G)** Western blot of cleaved-caspase 3, caspase 3, p-p65, and p65.

Moreover, as shown in Figures 2G,H, metformin dramatically increased the sensitivity of lung cancers to EGFR TKIs (G+M vs. G or O+M vs. O), especially of HCC827GR and HCC827OR cells, indicating that metformin overcame acquired resistance to EGFR TKIs. Similar results were observed using parental/sensitive PC9 and PC9OR cells (Figures S4A,B). As expected, gefitinib (G), osimertinib (O), metformin (M), or a combination of G+M or O+M did not have any significant effects on the proliferation of 16HBE cells (Figures S4C,D). To extend our findings to *in vivo*, HCC827GR cells were injected subcutaneously into both flanks of nude mice. When solid tumor reached an average volume of 200 mm^3^, mice were treated with gefitinib (12.5 mg/kg) alone (G), metformin (200 mg/kg) alone (M), or G+M daily by oral gavage. As shown in Figures 3A–C and Figure S5A, G+M dramatically inhibited tumor growth (Figures 3A,B) and promoted apoptosis of tumor compared to single treatment (Figure 3C and Figure S5A). As expected, p-p65 levels were slightly decreased in HCC827GR-xenograft tumors treated with G while non-detectable in those treated with M or G+M (Figure 3C and Figure S5A). Next, we examined whether metformin could delay the emergence of acquired resistance to EGFR TKIs, which was assessed by *in vitro* and *in vivo* models. First, a total of 500 viable HCC827 cells were seeded per well in a 96-well plate and treated with G, O, M, G+M, or O+M, and the wells of >50% confluence were scored as positive weekly (22). Gefitinib- and osimertinib-resistant colonies were found to begin to appear within 1 week (Figure 3D, G or O). Metformin (M) did not significantly delay the emergence of resistance alone while G+M or O+M dramatically delayed the emergence and reduced the incidence of resistant colonies. Next, nude mice harboring HCC827-xenograft tumors were treated with gefitinib (12.5 mg/kg, G), metformin (200 mg/kg, M), or combination (G+M) daily by gavage. During the treatment, metformin alone (M) slightly inhibited tumor growth while gefitinib alone (G) or G+M led to significant regression of xenograft tumor (Figures 3E,F). However, G was unable to prevent tumor regrowth after 4–5 weeks of treatment, suggesting emergence of acquired resistance, while G+M effectively suppressed tumor growth up to about 8 weeks (Figures 3E,F). Moreover, G+M dramatically promoted apoptosis compared to single drug treatment by significantly increasing the level of cleaved-caspase 3 in xenograft tumors (Figure 3G and Figure S5B). As expected, p-p65 levels were elevated in HCC827-xenograft tumors treated with G while significantly decreased in those treated with M and non-detectable in those treated with G+M, which was consistent with the results from the *in vitro* model.

### Metformin Suppressed Cancer Stemness of Lung Cancer

**Figure 4:**
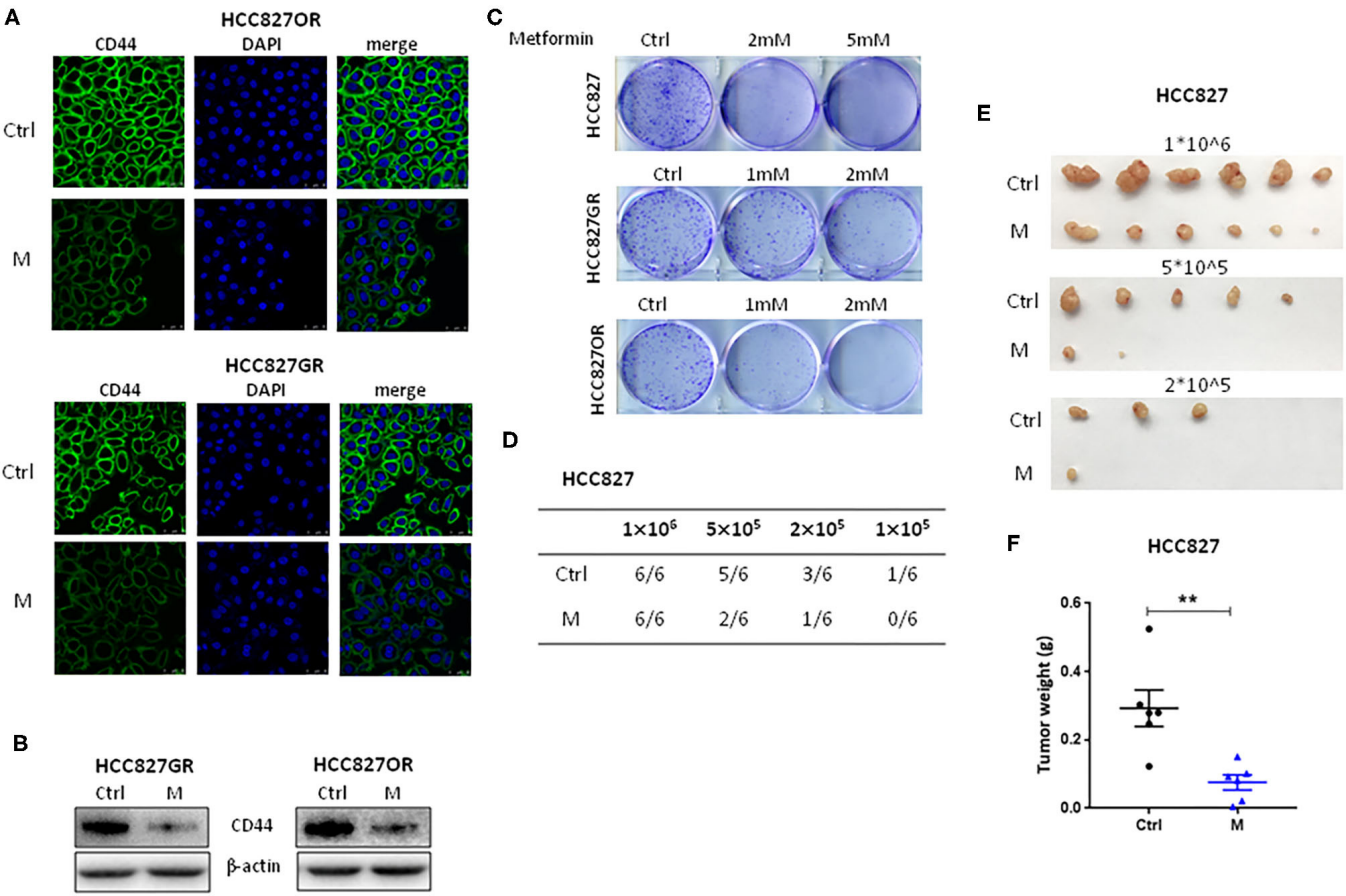
Metformin suppressed lung cancer stemness. **(A,B)** HCC827GR and HCC827OR cells were treated with 1 mM metformin (M) for 72 h. CD44 levels were analyzed by immunofluorescence staining **(A)** and Western blot **(B)**. **(A)** Nuclei were stained with DAPI. Scale bar, 50 μm. **(C)** Colony formation assay of indicated cells treated with indicated concentrations of metformin. **(D–F)** Limiting dilution transplantation assay. HCC827 cells were treated with 2 mM metformin (M) for 12 h prior to transplantation and then indicated viable cells were transplanted subcutaneously into nude mice. The incidence **(D)**, the photographs of tumors **(E)**, and the weight of tumors formed from 1 × 10^6^ HCC827 cell transplantation **(F)**. Results show the means ± SEM (*n* = 6, Student's *t*-test, **P < 0.01).

NF-κB signaling pathway has been documented to be involved in cancer stemness regulation. Acquisition of therapy resistance is partially associated with cancer stemness. Therefore, we next sought to evaluate the effects of metformin on lung cancer stemness. EGFR TKI-acquired resistant HCC827GR and HCC827OR cells were treated with metformin (M) and then the expression levels of CSC markers CD44 were examined by immunofluorescence (Figure 4A) and Western blot analysis (Figure 4B and Figure S5C). As shown in Figures 4A,B, the levels of CD44 in HCC827GR and HCC827OR cells were dramatically downregulated by M compared to control (Ctrl). Moreover, colony formation assay demonstrated that metformin suppressed self-renewal abilities of both sensitive and resistant cells *in vitro* (Figure 4C) in a concentration-dependent manner. To examine tumorigenic capacity *in vivo*, HCC827 cells were treated with metformin (M) for 12 h, and then viable cells (1 × 10^6^, 5 × 10^5^, 2 × 10^5^, or 1 × 10^5^) were implanted subcutaneously into nude mice. Metformin pretreatment led to a significant reduction in tumor incidence and tumor volume (Figures 4D–F). Overall, these results demonstrated that metformin suppressed lung cancer stemness.

### NF-κB Inhibition by Metformin Was Mediated by the AMPK/ERK Pathway

**Figure 5:**
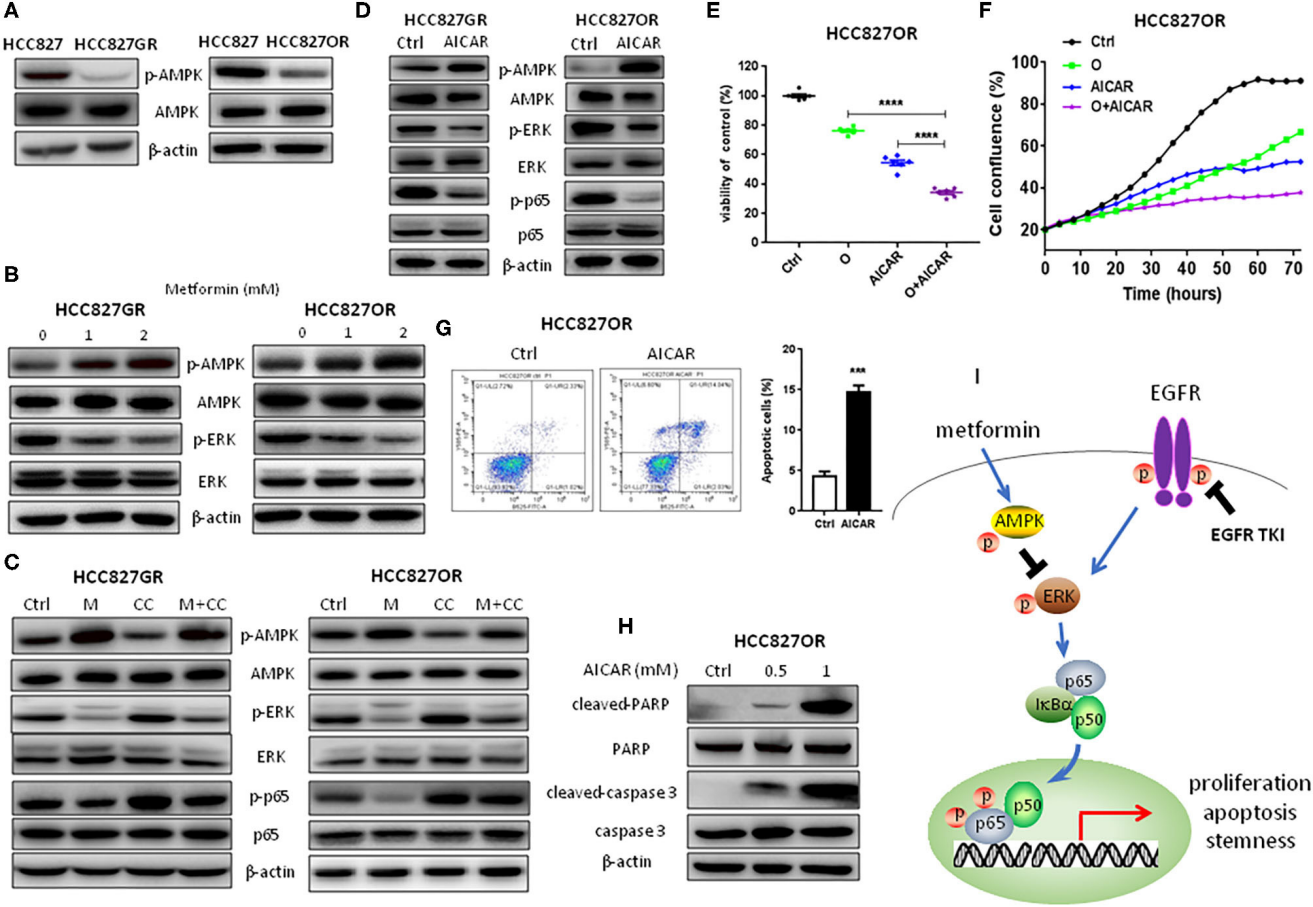
NF-κB inhibition by metformin was mediated by the AMPK/ERK pathway. **(A)** Western blot analysis of p-AMPK and AMPK in HCC827, HCC827GR, and HCC827OR cells. **(B)** Western blot analysis of p-AMPK, AMPK, p-ERK, and ERK in HCC827GR and HCC827OR cells treated with indicated concentrations (mM) of metformin for 72 h. **(C)** Western blot analysis of p-AMPK, AMPK, p-ERK, ERK, p-p65, and p65 in HCC827GR and HCC827OR cells treated with 2 mM metformin (M), 1 μM Compound C (CC), or M+CC for 72 h. **(D)** Western blot analysis of p-AMPK, AMPK, p-ERK, ERK, p-p65, and p65 in HCC827GR and HCC827OR cells treated with 1 mM AICAR for 72 h. **(E)** Cell viability by CCK8 assay of HCC827OR cells treated with 10 μM osimertinib (O), 0.5 mM AICAR, and O+AICAR for 72 h. Results show the means ± SEM (*n* = 6, one-way ANOVA, *P < 0.05, ****P < 0.0001). **(F)** IncuCyte growth curve of HCC827OR cells treated with 10 μM osimertinib (O), 0.5 mM AICAR, and O+AICAR. Results show the means ± SEM (*n* = 6). **(G)** Flow cytometry analysis of apoptosis of HCC827OR cells treated with 1 mM AICAR for 72 h (left panel) and quantitative analysis of apoptotic cells (right panel). Results show the means ± SEM (*n* = 3, Student's *t*-test, ***P < 0.001). **(H)** Western blot of cleaved-caspase 3, caspase 3, cleaved-PARP, and PARP in HCC827OR cells treated with indicated concentrations (mM) of AICAR for 72 h. **(I)** Schematic diagram depicting the mechanism underlying the effects of metformin in EFGR-mutant lung cancer.

At last, we investigated whether NF-κB inhibition was mediated by metformin-induced AMPK activation. First, AMPK activity between parental/sensitive HCC827 and EGFR TKI-acquired resistant HCC827GR and HCC827OR cells was compared. As shown in Figure 5A and Figure S6A, the expression level of p-AMPK was dramatically decreased in resistant cells compared with their parental/sensitive cells, demonstrating that AMPK activity was decreased in resistant cells. Then, resistant cells were treated with metformin and AMPK was found activated in a dose-dependent manner as shown in Figure 5B and Figure S6B. Meanwhile, ERK was also inhibited by metformin in a dose-dependent manner (Figure 5B and Figure S6B). To further demonstrate that NF-κB inhibition was mediated by metformin-induced AMPK activation, we used AMPK inhibitor Compound C (CC) to conduct rescue experiments. As shown in Figure 5C and Figure S6C, Compound C decreased p-AMPK levels leading to downstream ERK/NF-κB activation, which was abrogated by metformin. Moreover, AICAR was used to activate AMPK to examine whether activation of AMPK has similar effects to metformin. First, as shown in Figure 5D and Figure S7A, AMPK was activated by AICAR treatment and downstream ERK and p65 were inhibited concomitantly in EGFR TKI-acquired resistant HCC827GR and HCC827OR cells. Next, as expected, AICAR inhibited proliferation and promoted apoptosis of EGFR TKI-acquired resistant HCC827GR and HCC827OR cells (Figures 5E–H and Figures S7B, S8) as well as increased the sensitivity of resistant HCC827GR and HCC827OR to EGFR TKIs (Figures 5E,F and Figures S8A,B). Taken together, these data indicated that metformin activated AMPK to inhibit downstream ERK/NF-κB signaling, resulting in the effects on proliferation, apoptosis, and cancer stemness (Figure 5I).

## Discussion

NF-κB signaling plays a role in many aspects of cancer such as survival, metastasis, and therapy resistance (9, 11). During the course of EGFR TKI treatment, survival lung cancer cells became insensitive and acquired enhanced NF-κB activity concomitantly (12–14). Epidemiological studies have demonstrated that use of metformin has been associated with lowered cancer incidence in diabetic patients (23–28). Studies have also revealed the antitumor activities of metformin and plausible mechanisms *in vitro* and *in vivo* (29). One of them is its inhibitory effect on NF-κB signaling. Consistently, we found that metformin inhibited NF-κB activity and, as expected, suppressed proliferation and promoted apoptosis of EGFR-mutant lung cancer cells in a concentration-dependent manner. Interestingly, at the same time, we found that resistant cells were more sensitive to metformin than parental/sensitive cells in the aspect of proliferation and apoptosis while normal lung epithelial cells were insensitive to metformin, probably due to the differences in NF-κB activity levels in different cell lines (Figures 1A,B and Figure S1A). Moreover, metformin inhibited TNFα- and EGFR TKI-induced NF-κB activation (Figures 1H,I and Figures S1C,D). When combined with EGFR TKIs gefitinib or osimertinib, metformin had synergistic effects on the proliferation of lung cancer cells. More importantly, our data demonstrated that metformin significantly increased sensitivity of resistant lung cancers to EGFR TKIs and dramatically delayed acquired resistance emergence *in vitro* and *in vivo*. NF-κB signaling also plays an important role in cancer stemness regulation. As expected, we found that metformin suppressed lung cancer stemness in a concentration-dependent manner, with resistant cells more sensitive than parental/sensitive cells.

Next, we sought to determine whether metformin-induced NF-κB inhibition was AMPK-dependent. First, we found that AMPK activity was lowered in resistant cells and metformin activated AMPK as well as suppressed ERK activity in resistant cells in a concentration-dependent manner. Moreover, Compound C was used to inhibit AMPK activity and activate downstream ERK in resistant cells and metformin abrogated those Compound C-induced effects. Moreover, activation of AMPK by AICAR had similar effects to metformin. Taken together, we proposed that metformin could inhibit NF-κB p65 signaling by activating AMPK and inhibiting downstream ERK/ NF-κB to suppress lung cancer progression (Figure 5H).

In this study, the concentration of metformin used in *in vitro* experiments was higher than that observed in diabetic patients, in which the metformin plasma concentration is 6–30 μM. However, metformin intends to accumulate in tissues leading to a concentration several-fold higher than that in blood (30). This suggests that the concentration of metformin (1–5 mM) used in *in vitro* experiments might be attained in tumor tissue during cancer treatment. The dose of metformin used in *in vivo* experiments (200 mg/kg/day) is equivalent to 16.2 mg/kg/day in humans (31), which is much less than the maximum dose of 2,000 mg/day recommended by FDA for average adults. Therefore, the dose in this study is safe for human patients.

Our results identify metformin as a promising candidate for combinational therapy for lung cancer. Only few stage III clinical trials of metformin combined with chemotherapy, or targeted therapy to treat lung cancer patients have been conducted. We hope our study provides further molecular rationale and preclinical data to support combination of metformin with EGFR TKIs to treat lung cancer.

## Conclusions

In conclusion, using *in vitro* and *in vivo* EGFR-mutant lung cancer models with acquired resistance to EGFR TKIs, metformin was found to inhibit proliferation and promote apoptosis of EGFR-mutant lung cancer cells, especially acquired resistant cells. Moreover, metformin reversed and delayed acquired resistance to EGFR TKIs as well as suppressed cancer stemness in EGFR-mutant lung cancer. Mechanistically, those effects of metformin were associated with activation of AMPK, resulting in the inhibition of ERK/NF-κB signaling. Our data provided novel and further molecular rationale and preclinical data to support the combination of metformin with EGFR TKIs to treat EGFR-mutant lung cancer patients, especially with acquired resistance.

## Data Availability Statement

The raw data supporting the conclusions of this article will be made available by the authors, without undue reservation.

## Ethics Statement

The animal study was reviewed and approved by The Animal Care & Welfare Committee in Shanghai Jiao Tong University School of Medicine DLAS.

## Author Contributions

LL and LX designed the experiments and wrote the manuscript. LL, MH, TW, and YZ conducted the experiments and analyzed the results. LX and HC supervised the work and reviewed the manuscript. All authors have read and approved the final manuscript.

## Conflict of Interest

The authors declare that the research was conducted in the absence of any commercial or financial relationships that could be construed as a potential conflict of interest.

## Abbreviations

EGFR TKIs: epidermal growth factor receptor tyrosine kinase inhibitors
NSCLC: non-small cell lung cancer
NF-κB: nuclear factor-κB
EMT: epithelial-mesenchymal transition
PARP: poly ADP-ribose polymerase
AMPK: AMP-activated protein kinase
G: gefitinib
O: osimertinib
M: metformin
GR: gefitinib-resistant
OR: osimertinib-resistant
AICAR: 5-aminoimidazole-4-carboxamide-1-β-D-ribofuranoside.
