# Supplementary material for: Metformin Overcomes Acquired Resistance to EGFR TKIs in EGFR-Mutant Lung Cancer via AMPK/ERK/NF-κB Signaling Pathway
Source: Front Oncol. 2020 Sep 10;10:1605. doi: 10.3389/fonc.2020.01605 (PMC7511631; doi:10.3389/fonc.2020.01605)
Supplement: Supplementary file 1 [file Table_1.DOCX]

**Table S1. List of antibodies**

| **Table S2. Antibodies** | | | |
| --- | --- | --- | --- |
| **Target** | **Use** | **Vendor** | **Catalog #** |
| PARP | WB | Cell Signaling Tech | 9542 |
| Cleaved PARP | WB | Cell Signaling Tech | 5625 |
| Caspase-3 | WB | Cell Signaling Tech | 9665 |
| Cleaved Caspase-3 | WB | Cell Signaling Tech | 9664 |
| β-actin | WB | Santa Cruz Biotech | sc-47778 |
| NF-κB p65 | WB, Immunostaining | Cell Signaling Tech | 3195 |
| phospho-NF-κB p65 | WB | Cell Signaling Tech | 5741 |
| pAMPK | WB | Cell Signaling Tech | 2535 |
| AMPK | WB | Santa Cruz Biotech | 4461 |
| pERK | WB | Cell Signaling Tech | 4376 |
| ERK | WB | Cell Signaling Tech | 4695 |
| CD44 | Immunostaining | Cell Signaling Tech | 9585 |
