# Supplementary material for: Metformin Overcomes Acquired Resistance to EGFR TKIs in EGFR-Mutant Lung Cancer via AMPK/ERK/NF-κB Signaling Pathway
Source: Front Oncol. 2020 Sep 10;10:1605. doi: 10.3389/fonc.2020.01605 (PMC7511631; doi:10.3389/fonc.2020.01605)
Supplement: Supplementary file 2 [file Data_Sheet_1.PDF]

Figure S1. Quantification of Western blot in Figure 1.

(A) Quantification of Figure 1A. Densitometric analysis of western blot signals of p-p65 relative to 16HBE and normalized to loading control β-actin. (B-D) Quantification of Figure 1D (B), 1H(C) and 1I (D). Densitometric analysis of western blot signals of p-p65 relative to control and normalized to loading control β-actin. Results show means ± SEM (n=3, Student's t-test or one-way ANOVA, * P < 0.05, ** P < 0.01, *** P < 0.001, **** P < 0.0001).

Figure S2. NF-κB was activated in EGFR-mutant lung cancer PC9 cells with acquired resistance to EGFR TKIs

(A) Phosphorylated NF-κB p65 (p-p65) and NF-κB p65 (p65) in PC9, PC9GR and PC9OR cells were detected by western blot. (B) Quantification of (A). Densitometric analysis of western blot signals of p-p65 relative to PC9 and normalized to loading control β-actin. Results show means ± SEM (n=3, Student's t-test, *** P < 0.001, **** P < 0.0001).

Figure S3. Quantification of Western blot in Figure 2.

(A, B) Quantification of Figure 2A (A) and 2D (B). Densitometric analysis of western blot signals of cleaved-PARP and cleaved-caspase 3 relative to control and normalized to loading control β-actin. Results show means ± SEM (n=3, Student's t-test, * P < 0.05, ** P < 0.01, *** P < 0.001, **** P < 0.0001).

Figure S4. The effects of metformin on cell proliferation

(A) PC9 cells were treated with 10 nM osimertinib (O), 2 mM metformin (M), or O+M. IncuCyte growth curve (left panel) and cell viability by CCK8 assay (right panel, 72 hours). Results show the means ± SEM (n=6 for IncuCyte; n=4 for CCK8, one-way ANOVA, **** P < 0.0001). (B) PC9OR cells were treated with 10 μM osimertinib (O), 1 mM metformin (M) or O+M. IncuCyte growth curves (left panel) and cell viabilities by CCK8 assay (right panel, 72 hours). Results show the means ± SEM (n=6 for IncuCyte; n=6 for CCK8, one-way ANOVA, **** P < 0.0001). (C, D) 16HBE cells were treated with 10 nM osimertinib (O), 10 nM gefitinib (G), 2 mM metformin (M), O+M or G+M. IncuCyte growth curve (A) and cell viability by CCK8 assay (B, 72 hours). Results show the means ± SEM (n=5-6).

Figure S5. Quantification of Western blot in Figure 3 and 4.

(A, B) Quantification of Figure 3C (A) and 3G (B). Densitometric analysis of western blot signals of cleaved-caspase 3 and p-p65 relative to control and normalized to loading control β-actin. Results show means ± SEM (n=4, one-way ANOVA, * P < 0.05, ** P < 0.01, **** P < 0.0001). (C) Quantification of Figure 4B. Densitometric analysis of western blot signals of CD44 relative to control and normalized to loading control β-actin. Results show means ± SEM (n=3, Student's t-test, *** P < 0.001).

Figure S6. Quantification of Western blot in Figure 5A-5C.

(A) Quantification of Figure 5A. Densitometric analysis of western blot signals of p-AMPK relative to HCC827 and normalized to loading control β-actin. Results show means ± SEM (n=3, Student's t-test, ** P < 0.01). (B) Quantification of Figure 5B. Densitometric analysis of western blot signals of p-AMPK and p-ERK relative to control and normalized to loading control β-actin. Results show

means ± SEM (n=3, Student's t-test, * $P < 0.05$, ** $P < 0.01$, *** $P < 0.001$). (C) Quantification of Figure 5C. Densitometric analysis of western blot signals of p-AMPK, p-ERK and p-p65 relative to control and normalized to loading control β-actin. Results show means ± SEM (n=3, one-way ANOVA, * $P < 0.05$, ** $P < 0.01$, *** $P < 0.001$).

Figure S7. Quantification of Western blot in Figure 5D and 5H.
(A) Quantification of Figure 5D. Densitometric analysis of western blot signals of p-AMPK, p-ERK and p-p65 relative to control and normalized to loading control β-actin. Results show means ± SEM (n=3, Student's t-test, * $P < 0.05$, ** $P < 0.01$, *** $P < 0.001$). (B) Quantification of Figure 5H. Densitometric analysis of western blot signals of cleaved-caspase 3 and cleaved-PARP relative to control and normalized to loading control β-actin. Results show means ± SEM (n=3, Student's t-test, * $P < 0.05$, ** $P < 0.01$, *** $P < 0.001$).

Figure S8. The effects of AICAR on HCC827GR cells
(A) Cell viability by CCK8 assay of HCC827GR cells treated with 10 μM gefitinib (G), 0.5 mM AICAR, and G+AICAR for 72 hours. Results show the means ± SEM (n=6, one-way ANOVA, *** $P < 0.001$). (B) IncuCyte growth curve of HCC827GR cells treated with 10 μM gefitinib (G), 0.5 mM AICAR, and G+AICAR. Results show the means ± SEM (n=6). (C) Flow cytometry analysis of apoptosis of HCC827GR cells treated with 1 mM AICAR for 72 hours (upper panel) and quantitative analysis of apoptotic cells (lower panel). Results show the means ± SEM (n=3, Student's t-test, **** $P < 0.0001$). (D) Western blot of cleaved-caspase 3, caspase 3, cleaved-PARP and PARP in HCC827GR cells treated with indicated concentration of AICAR (mM) for 72 hours. (E) Quantification of (D). Densitometric analysis of western blot signals of cleaved-caspase 3 and cleaved-PARP relative to control and normalized to loading control β-actin. Results show means ± SEM (n=3, Student's t-test, ** $P < 0.01$, *** $P < 0.001$, **** $P < 0.0001$).
