# Supplementary figures and images for: Metformin Overcomes Acquired Resistance to EGFR TKIs in EGFR-Mutant Lung Cancer via AMPK/ERK/NF-κB Signaling Pathway
Source: Front Oncol. 2020 Sep 10;10:1605. doi: 10.3389/fonc.2020.01605 (PMC7511631; doi:10.3389/fonc.2020.01605)

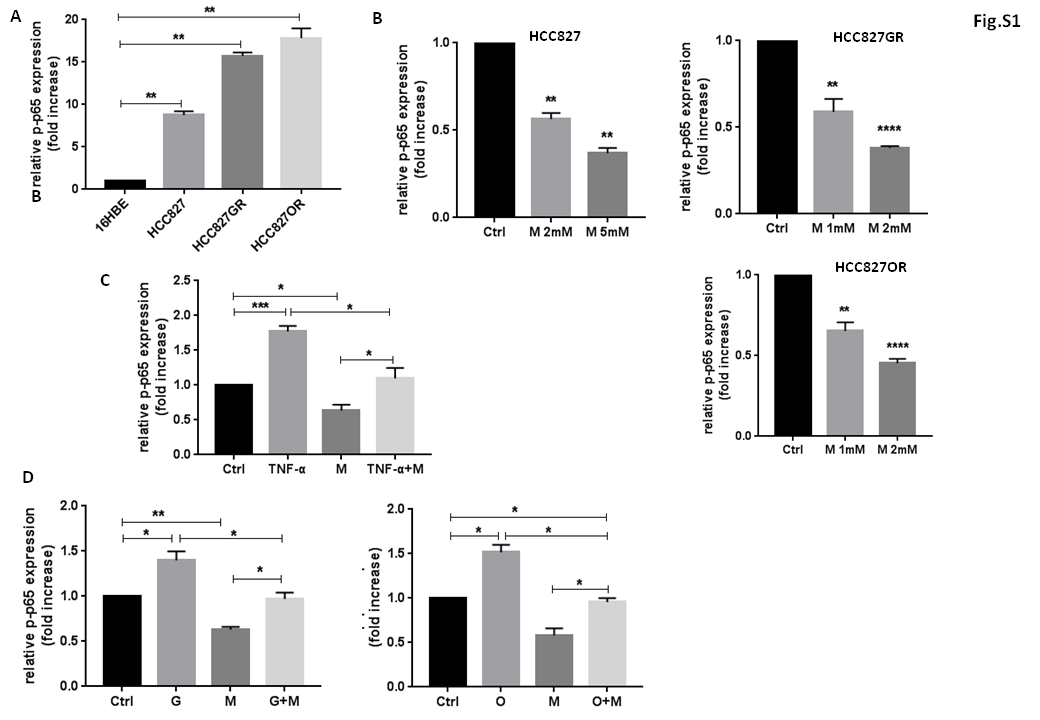

Supplement: Supplementary file 3 [file Image_1.TIF]

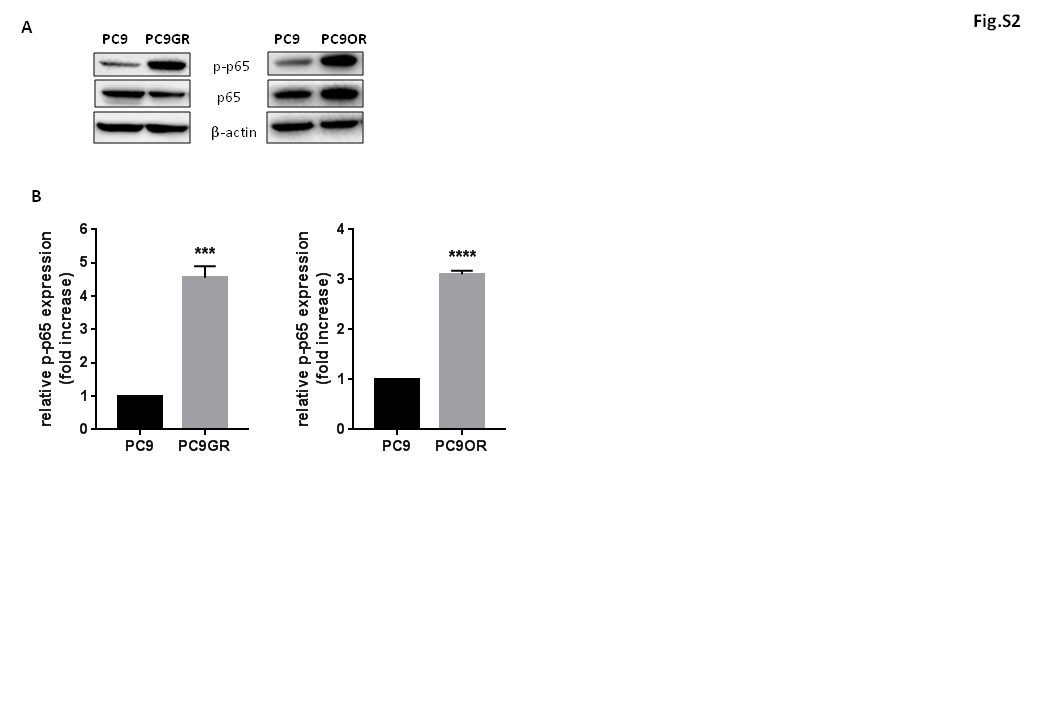

Supplement: Supplementary file 4 [file Image_2.TIF]

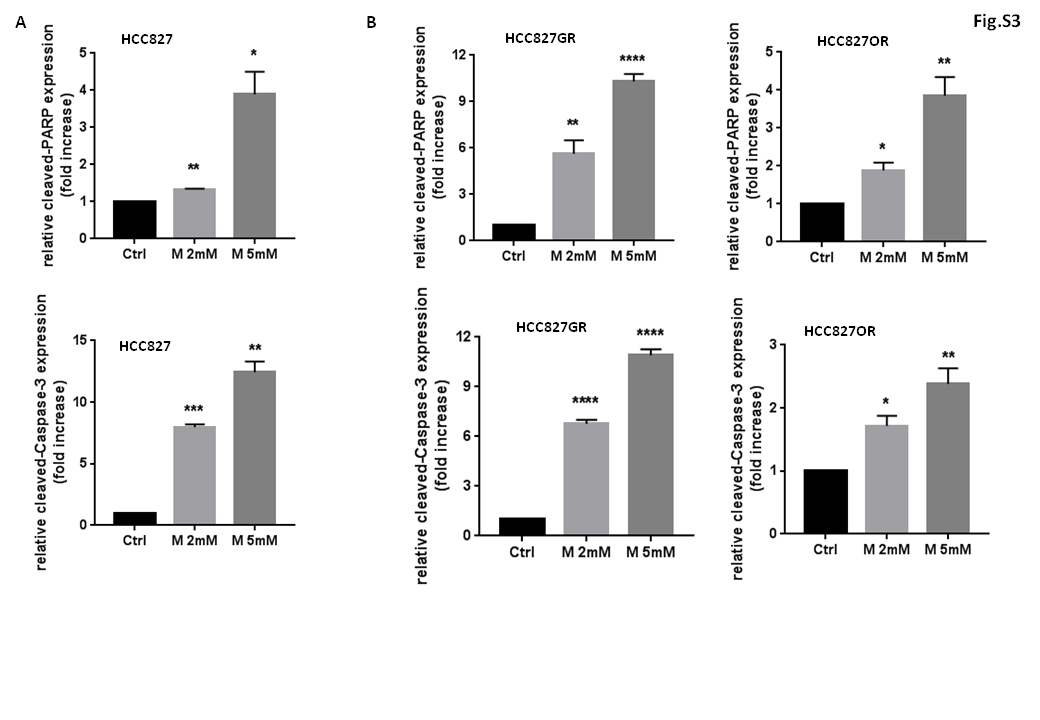

Supplement: Supplementary file 5 [file Image_3.TIF]

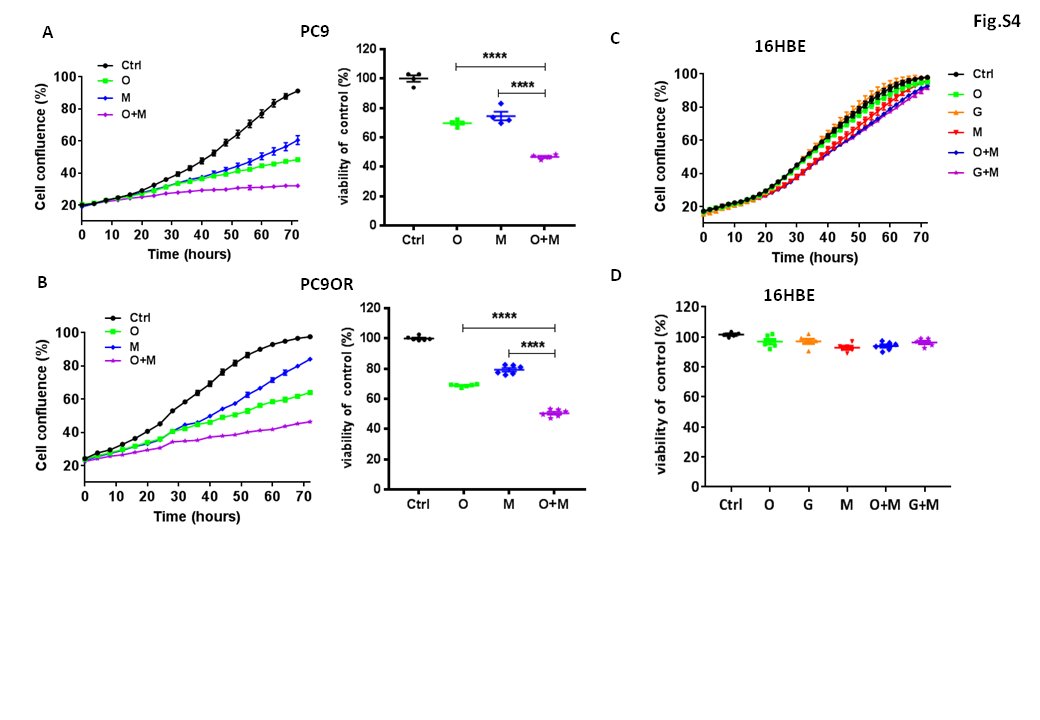

Supplement: Supplementary file 6 [file Image_4.TIF]

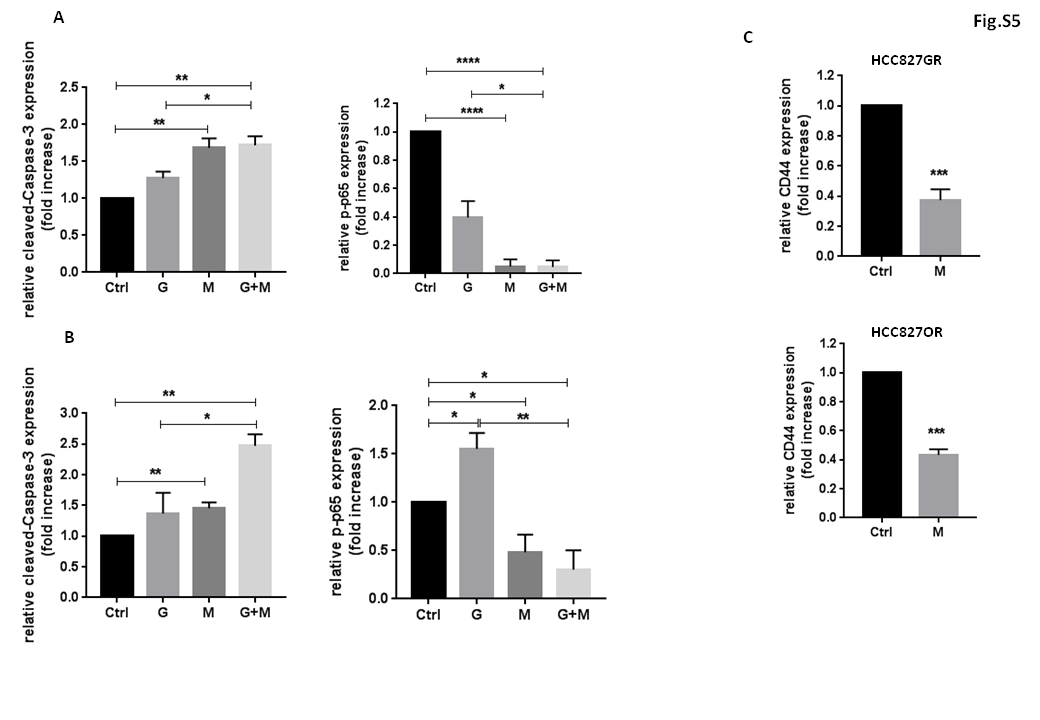

Supplement: Supplementary file 7 [file Image_5.TIF]

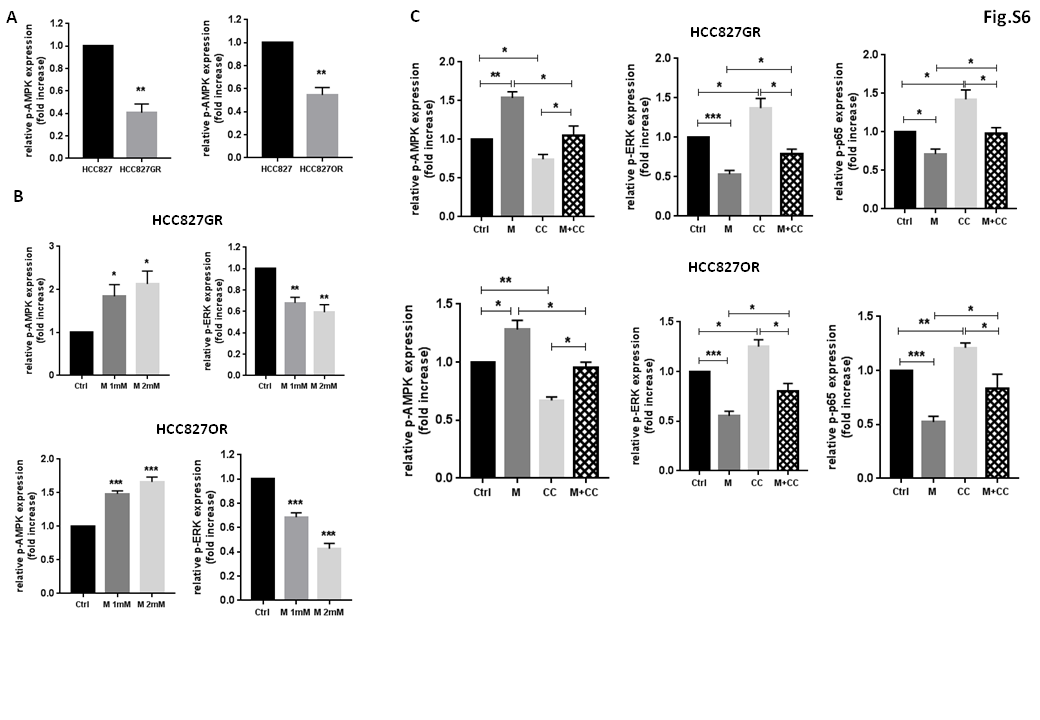

Supplement: Supplementary file 8 [file Image_6.TIF]

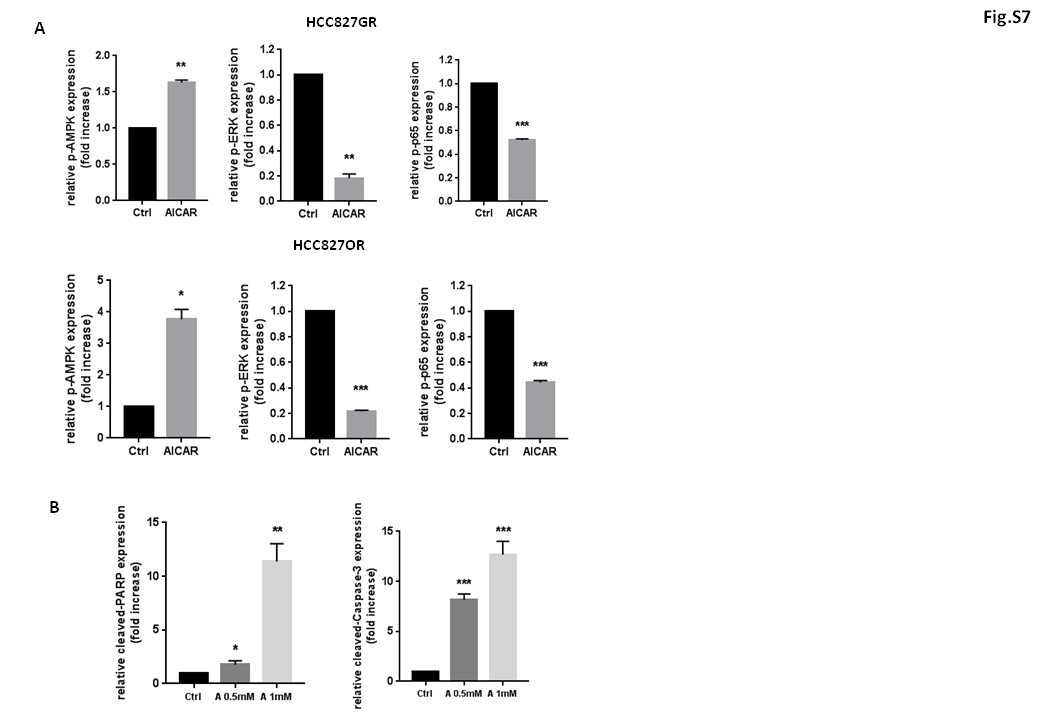

Supplement: Supplementary file 9 [file Image_7.TIF]

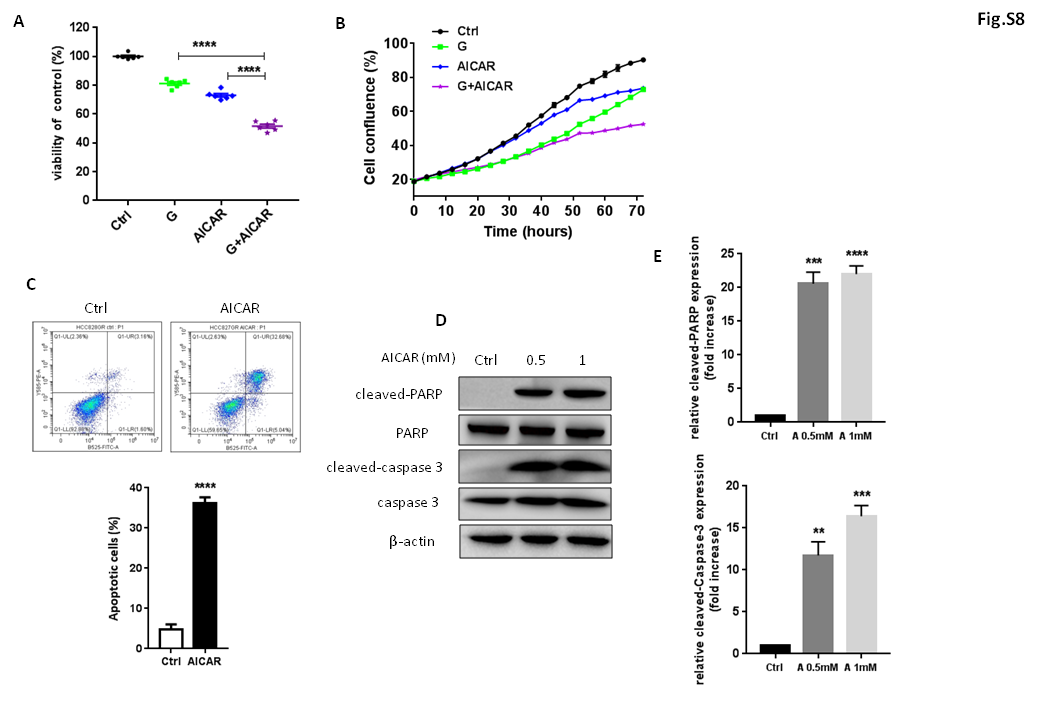

Supplement: Supplementary file 10 [file Image_8.TIF]
